# Efficacy of the PanCareFollowUp eHealth Lifestyle Intervention for Survivors of Childhood, Adolescent and Young Adult Cancer

**DOI:** 10.1002/cam4.70694

**Published:** 2025-02-28

**Authors:** Selina R. van den Oever, Eline Bouwman, Helena J. H. van der Pal, Philippa C. Steensma, Vera Araujo‐Soares, Morven Brown, Tomas Kepak, Katerina Kepakova, Marta Fiocco, Lucy M. M. Fremouw, Maria M. W. Koopman, Raphaële R. L. van Litsenburg, Patrick van der Torre, Joyce Wilbers, Roderick Skinner, Leontien C. M. Kremer, Jacqueline Loonen, Saskia M. F. Pluijm, the PanCareFollowUp consortium

**Affiliations:** ^1^ Princess Máxima Center for Pediatric Oncology Utrecht the Netherlands; ^2^ Radboud University Medical Centre Nijmegen the Netherlands; ^3^ Center for Preventive Medicine and Digital Health Medical Faculty Mannheim, Heidelberg University Heidelberg Germany; ^4^ Wolfson Childhood Cancer Research Centre Newcastle UK; ^5^ International Clinical Research Center, St. Anne's University Hospital Brno Brno Czech Republic; ^6^ Mathematical Institute Leiden University Leiden the Netherlands; ^7^ Department of Biomedical Data Science, Section Medical Statistics Leiden University Medical Center Leiden the Netherlands; ^8^ Great North Children's Hospital, and Translational and Clinical Research Institute, and Centre for Cancer Newcastle University Newcastle UK; ^9^ University Medical Center Utrecht, Wilhelmina Children's Hospital Utrecht the Netherlands

**Keywords:** CAYA cancer survivorship, eHealth, lifestyle, lifestyle intervention, paediatric oncology

## Abstract

**Purpose:**

A healthy lifestyle may prevent or mitigate late effects in childhood, adolescent and young adult (CAYA) cancer survivors. To support survivors in adopting healthier behaviours, the PanCareFollowUp (PCFU) Lifestyle intervention was developed, encompassing 4 months of online lifestyle coaching aimed at achieving a personal lifestyle goal. The aims of this study were to (1) determine the efficacy of this intervention on lifestyle outcomes over time and (2) identify predictors for goal achievement.

**Patients and Methods:**

Fifty‐eight survivors were enrolled. Outcomes were assessed at baseline (T0), after 4 months of coaching (T1) and after 4 months of follow‐up (T2). The primary outcome included the percentage of survivors successful in achieving and sustaining their goal, whereas secondary outcomes included differences in body mass index (BMI), diet and physical activity. To evaluate the adjusted, longitudinal effects on secondary outcomes, linear mixed models were estimated. Predictors for goal achievement were identified through logistic regression analysis.

**Results:**

At T1 and T2, 68% and 76% of goals were achieved or sustained, respectively. Mean differences between T2 and T0 showed significant improvements in BMI (−0.5 kg/m^2^), diet (−0.6 points) and physical activity (+7.7 h/week). Estimation of multivariable models also showed positive effects. Participants with a lower BMI and fewer depressive feelings at baseline were more likely to achieve and/or sustain their goals at T2.

**Conclusion:**

Findings suggest that the PCFU Lifestyle intervention supports survivors in making lifestyle changes. Results can be used to inform a subsequent randomised intervention study and integrate lifestyle coaching into care.

**Trial Registration:** International Clinical Trial Registry Platform (ICTRP) number: NL8932 (ICTRP Search Portal [who. int]). Registered on 29 September 2020

## Graphical abstract

Within the European PanCareFollowUp project, an eHealth lifestyle intervention was developed to guide survivors of childhood, adolescent and young adult cancer in adopting healthier behaviours. Results from this efficacy analysis are positive and can be used for further study and implementation.

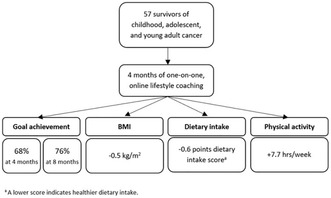

**Abbreviations**
BMI: body mass index
CAYA: childhood, adolescent and young adult
DFHQ: Dutch Food Habit Questionnaire
GSE: general self‐efficacy
PanCare: Pan‐European network for care of survivors after childhood and adolescent cancer
PCFU: PanCareFollowUp
RICk: readiness, importance, confidence and knowledge
SD: standard deviation
SeMaS: Self‐Management Screening
SQUASH: Short Questionnaire to Assess Health‐Enhancing physical activity

## 1 Introduction

Over the past decades, the treatment of childhood, adolescent and young adult (CAYA) cancer has greatly improved, resulting in a considerable increase in the 5‐year survival rate, which is now over 80% [1]. Nevertheless, despite these positive advances, three out of four CAYA cancer survivors today still suffer from one or more long‐term physical, mental and/or social health problems, such as cardiovascular disease, second malignancies, anxiety, chronic fatigue and lower socioeconomic status [2, 3, 4, 5, 6]. To reduce these health risks and improve or maintain CAYA cancer survivors' quality of life, adopting and sustaining healthy lifestyle behaviours is essential [7, 8, 9].

Making positive, sustainable lifestyle changes is challenging for most individuals, in particular for CAYA cancer survivors when dealing with physical or mental barriers related to their cancer history. Given that each survivorship journey is highly personal, CAYA cancer survivors may greatly benefit from a tailored lifestyle intervention with a person‐centred approach, such as one‐on‐one, individualised lifestyle coaching. In the Dutch general population, the effectiveness of combined lifestyle interventions—comprising lifestyle coaching, health education and peer support—has been well documented [10, 11]. Nevertheless, scientific evidence solely describing the effects of (individualised) lifestyle coaching remains scarce, and no lifestyle coaching interventions have been developed and evaluated for the cancer patient or survivor population. Therefore, as part of the PanCareFollowUp (PCFU) project (www.pancarefollowup.eu) [12], which was funded by the European Union, a person‐centred, eHealth lifestyle coaching intervention was developed. This intervention was designed to support CAYA cancer survivors in achieving a personal lifestyle goal and improve body mass index (BMI), dietary intake, physical activity, self‐efficacy, and self‐management skills, while limiting time constraints and travel burden [13].

The primary aim of this study was to determine the efficacy of the PCFU Lifestyle intervention on lifestyle behaviours in CAYA cancer survivors, including achieving and sustaining a personal lifestyle goal, BMI, dietary intake and physical activity. A secondary aim was to identify possible predictors associated with personal lifestyle goal achievement and sustainability upon receiving the intervention.

## 2 Methods

### 2.1 The PCFU Lifestyle Intervention

A comprehensive description of the PCFU Lifestyle intervention and methodology to evaluate its feasibility and efficacy is provided by Bouwman et al. [13]. The results of the feasibility evaluation will be described elsewhere (unpublished manuscript).

**FIGURE 1:**
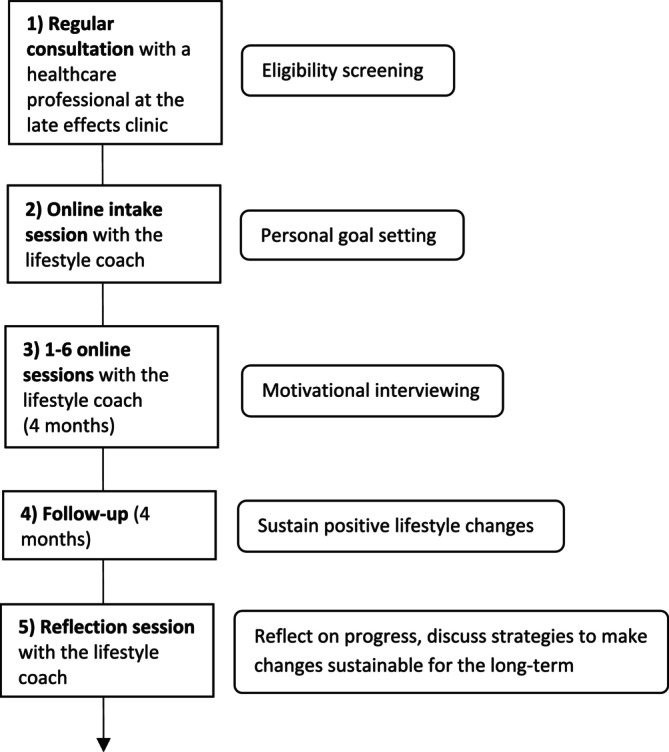
Flow chart of the PCFU Lifestyle intervention.

The PCFU Lifestyle intervention was based on the Transtheoretical Model or Stages of Change Model, in which people are guided to move through five stages of change when changing behaviour (precontemplation, contemplation, preparation, action and maintenance). Specifically, the PCFU Lifestyle intervention consisted of an online intake session followed by one to six regular online coaching sessions over the course of 4 months (Figure 1). Coaching was provided individually by a certified lifestyle coach specialised in CAYA cancer survivorship. The exact number of sessions, as well as timing and duration, was tailored to the survivor's needs and preferences.

Prior to the start of the regular coaching sessions, an intake session took place where the CAYA cancer survivor and lifestyle coach could get acquainted. In addition, during this session, the survivor was asked to set a personal lifestyle goal related to weight loss, a healthier diet and/or more physical exercise. This was the goal that, during the subsequent 4 months, the survivor would aim to achieve. The lifestyle coach guided the survivor in setting a goal that was both realistic and clinically relevant.

To support the survivor in moving through each stage of change, each coaching session started by exploring the survivor's current stage of change, followed by motivational interviewing by the lifestyle coach. Motivational interviewing integrates elements from multiple theories, including the Transtheoretical Model, the Self‐Determination Theory, Cognitive Dissonance Theory, and the Social Learning Theory.

After the 4‐month coaching period, survivors were followed up for another 4 months, after which a reflection session was scheduled to discuss the survivors' progress and ability to sustain the accomplished lifestyle improvements. In addition, this reflection session was used to discuss strategies to make the lifestyle changes sustainable for the long term.

### 2.2 Participants

CAYA cancer survivors were enrolled during routine clinic consultations at the Princess Máxima Center and Radboud University Medical Center between December 2020 and June 2023. Through sample size calculations, it was determined that 52 participants should yield sufficient statistical power (80%) at a significance level of 0.05, facilitating the detection of medium effects across all outcomes (Appendix S2).

Eligible participants were aged 16–55 years at enrolment, were diagnosed with any type of cancer before the age of 25 years, were at least 5 years post‐treatment and were cancer‐free at the time of the study. In addition, participants were eligible if they were either overweight or obese (BMI ≥ 25 kg/m^2^) and/or did not meet the WHO guideline for physical activity [14]. Lastly, participants had to be sufficiently motivated to adopt healthier lifestyle behaviours, which were assessed using a shortened version of the RICk (readiness, importance, confidence and knowledge) assessment [15].

Survivors were excluded from this study if they met at least one of the following criteria: diagnosed with Down syndrome, cognitive disorders, severe depressive symptoms and/or complex endocrine disorders; already participating in an intervention study or other interventions aiming to improve lifestyle behaviours; having a BMI ≤ 20 kg/m^2^ or having severe physical limitations that may hinder proper participation in the intervention. Participants who failed to complete baseline measurements or the online intake session were not included in this analysis.

### 2.3 Data Collection Instruments

Upon data collection, we made use of the cloud‐based clinical data management platform Castor Electronic Data Capture (Castor EDC), which complies with the General Data Protection Regulation (GDPR). During each coaching session, the survivor's progress was captured in a comprehensive coaching report. In addition, participants were asked to complete online questionnaires and wear an accelerometer (Yamax Digiwalker SW‐200) for 7 consecutive days at three different time points: before the intake session (T0, baseline measurement), after the last regular session (T1, 4 months after baseline) and after the reflection session (T2, 8 months after baseline). The reliability of the Yamax Digiwalker and its suitability for scientific research were determined by Kooiman et al. [16].

### 2.4 Covariates

Information about socio‐demographics, including living situation, educational attainment, and employment status, was obtained using the online questionnaire at T0. In addition, diagnosis and treatment‐related data were extracted from electronic medical records.

### 2.5 Outcome Measures

The primary outcome measure encompassed the percentage of participants able to achieve and sustain their personal lifestyle goal at T1 and T2. Personal lifestyle goals were defined by the survivor and lifestyle coach through shared‐decision making and were related to weight loss, a healthier diet, and/or more physical exercise. Whether the lifestyle goal, as defined by the survivor and lifestyle coach, was actually achieved was assessed by the lifestyle coach during each regular coaching session and the reflection session.

Secondary outcomes included the differences in BMI, dietary intake, physical activity, self‐efficacy and self‐management skills between T2 and T0. To calculate BMI, questionnaires inquired about participants' weight, whereas height was extracted from the electronic medical records. The Dutch Food Habit Questionnaire (DFHQ) was used to obtain information about participants' diet, encompassing fat intake (score 12–60), vegetable and fruit intake (score 5–21) and fibre intake (score 0–8). A higher score indicated a greater intake in the respective category. Subsequently, from these components, a total dietary intake score was derived, ranging from 3 to 9 (1–3 points allocated per category, with a score of 1 signifying a healthier intake). A lower total dietary intake score represented a healthier dietary intake. To assess physical activity, we made use of the accelerometer and validated Short Questionnaire to Assess Health‐Enhancing physical activity (SQUASH), which assessed the time spent on commuting activities, physical activity at work, household activities and activities in leisure time. In addition, it allowed for the calculation of a physical activity score (total SQUASH score), which accounted for the duration of each physical activity, as well as its self‐reported intensity (light/moderate/vigorous) and predefined metabolic equivalent (MET) score.

Self‐efficacy was assessed by making use of the General Self‐Efficacy (GSE) Scale [17]. This scale included 10 statements about behavioural responses or actions, whose applicability had to be rated on a 4‐point Likert scale. A total self‐efficacy score, ranging from 10 to 40, was calculated by adding up scores of the individual statements. A higher score suggested stronger self‐efficacy. To assess self‐management skills, we made use of six domains of the Self‐Management Screening (SeMaS) tool [18], namely, (1) the burden of late effects on participants' daily life (score 0–10, *no burden* to *unbearable burden*), (2) willingness to self‐manage (score 0–3, *not willing* to *willing*), (3) perceived control over health (score 0–6, *no control* to *a lot of control*), (4) confidence in the ability to sustain a healthy lifestyle (score 0–6, *low confidence* to *high confidence*), (5) feelings of anxiety (score 0–8, *never* to *often*), and (6) feelings of depression (score 0–6, *never* to *often*).

### 2.6 Statistical Analyses

First, descriptive statistics were calculated for participant characteristics and secondary outcomes at baseline. Categorical variables are reported as absolute numbers and percentages. For continuous data, means and standard errors are shown. Adherence to the intervention was defined as completion of the intake session, at least 1 of 6 regular sessions and the reflection session 8 months after baseline. Adherence to the study was defined as completion of the self‐reported measurements (questionnaires and accelerometer) at T0, T1, and T2. We assessed the comparability of the descriptives at baseline between participants who completed the questionnaires and those who did not.

To analyse the intervention's effect on the primary outcome of the study, percentages were calculated for participants who reached their predefined lifestyle goal at T1 and those who achieved or sustained their goal at T2. To determine the intervention's effect on secondary outcome measures, the mean differences between T2 and T0 (∆T2 − T0) were examined for complete cases using a paired *t*‐test (normally distributed data), Wilcoxon signed‐rank test (non‐normally distributed data), and Fischer's exact test (categorical data). Due to the presence of three repeated measurements, linear mixed models were estimated to study the adjusted effects of the intervention on self‐reported BMI, dietary intake and physical activity. Based on prior knowledge, age, age at primary cancer diagnosis, sex, educational attainment and late effects burden were incorporated in these models. Additional covariates were selected based on the associations with the outcomes in univariable analysis (*p* < 0.2). In this step, all collected variables were investigated, and those with an adjusted *p*‐value < 0.05 were retained in the final models. In addition, the model for physical activity was adjusted for the timing of questionnaire completion (before, during or after COVID‐19 restrictions employed from 1 March 2020 till 15 March 2022) as these restrictions influenced the level of physical activity in the general population [19]. Lastly, assumptions for linear mixed modelling were checked, and to account for non‐normality in the residuals, the response variable for physical activity was transformed using a natural logarithm. The estimated regression coefficients were transformed to the original scale by taking the exponential transformation.

To identify possible predictors for the attainment and sustainability of personal lifestyle goals during the follow‐up period, a multivariable logistic regression analysis model was estimated. Covariates were selected as described above for the linear mixed modelling analysis. Potential predictors for greater improvements in BMI, dietary intake, and physical activity (such as the number of coaching sessions and the type of personal lifestyle goal) were assessed in an extended analysis presented in Appendix S3. For all analyses, two‐sided *p*‐values < 0.05 were considered statistically significant. All statistical analyses were performed using SPSS version 26 (Armonk, NY: IBM Corporation).

## 3 Results

### 3.1 Participants

**FIGURE 2:**
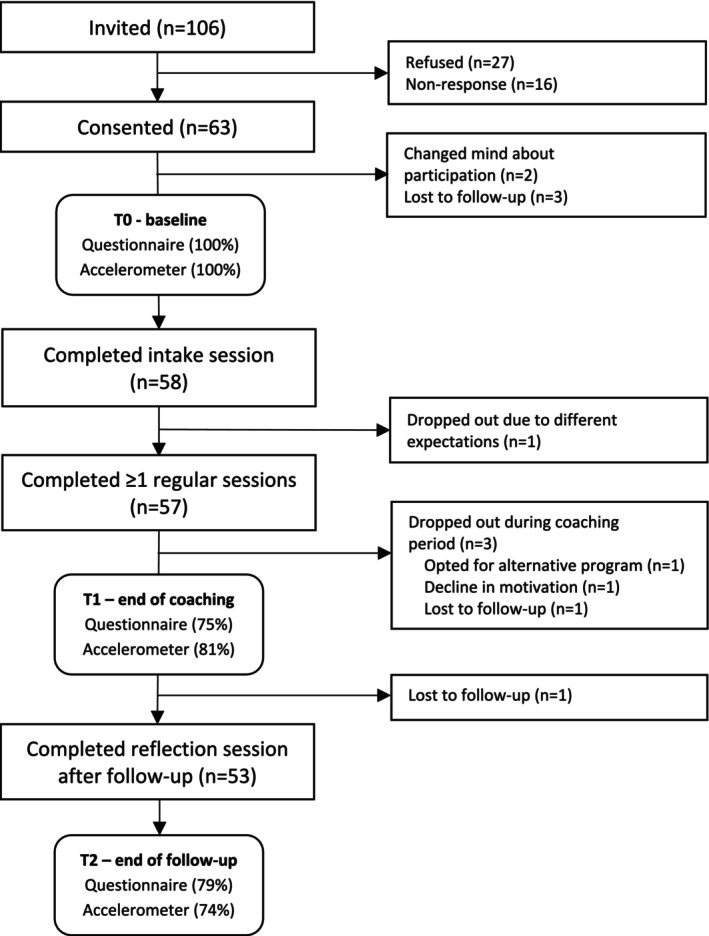
Study flow and participation.

In total, 106 CAYA cancer survivors were invited to participate in this study, of which 63 provided informed consent (Figure 2). Subsequently, 58 survivors (92%) entered the study after completion of the baseline measurements and intake session with the lifestyle coach. Of these 58 participants, 57 (98%) completed at least one regular coaching session, 53 participants (91%) fully adhered to the intervention (i.e., completed the intake, at least one regular session and the reflection session), and 5 participants (9%) dropped out.

The primary outcome measure, namely the attainment and sustainability of personal lifestyle goals, could be assessed for 57 participants at T1 and for 53 participants at T2. For the secondary outcomes, 43/57 (75%) participants completed ≥ 50% of the questionnaires at T1 and 42/53 (79%) at T2. The accelerometer was worn for 7 consecutive days and returned by 46/57 (81%) participants at T1 and 39/53 (74%) participants at T2.

Participant characteristics and lifestyle behaviours at baseline are provided in Table 1. No differences between questionnaire completers and non‐completers were identified, except for older age at baseline evaluation for T1 questionnaire completers (35.7 years vs. 28.7 years, respectively; *p* = 0.04) and a healthier dietary intake at baseline for those who completed the questionnaires at T2 (overall diet score of 6.2 vs. 7.3, respectively; *p* = 0.01).

**TABLE 1:** Characteristics at baseline.

Characteristic	Total (*n* = 58)	Characteristic	Total (*n* = 58)
	*n* (%)		*n* (%)
Sex		Educational attainment	
Male	25 (43.1)	Low	9 (15.5)
Female	33 (56.9)	Middle	32 (55.2)
Age		High	17 (29.3)
Mean (SD)	33.9 (11.5)	Employment status	
16–29	23 (39.7)	Paid employment	37 (63.8)
30–39	15 (25.9)	Unpaid employment	2 (3.4)
≥ 40	20 (34.5)	Student	11 (19.0)
Age at primary cancer diagnosis		Unemployed, receiving benefits	3 (5.2)
Mean (SD)	9.3 (5.8)	Unemployed, not receiving benefits	5 (8.6)
Primary cancer diagnosis		Late effects burden (0–10)	
Leukaemia	21 (36.2)	Mean (SD)	3.6 (2.6)
Non‐Hodgkin lymphoma	7 (12.1)	Feelings of anxiety (0–8)	
Hodgkin lymphoma	10 (17.2)	Mean (SD)	0.6 (1.1)
CNS tumour	7 (12.1)	Feelings of depression (0–6)	
Neuroblastoma	5 (8.6)	Mean (SD)	0.8 (1.1)
Bone tumour	4 (6.9)	BMI (kg/m^2^)	
Other^a^	4 (6.9)	Mean (SD)	30.5 (4.9)
Cancer treatment^b^		< 25 kg/m^2^ (healthy)	4 (6.9)
Surgery	18 (31.0)	25–30 kg/m^2^ (overweight)	27 (46.6)
Chemotherapy	52 (89.7)	> 30 kg/m^2^ (obese)	27 (46.6)
Radiotherapy	24 (41.4)	Dietary intake	
Stem cell transplantation	3 (5.2)	Overall diet score (3–9), mean (SD)^c^	6.5 (1.5)
Comorbidities		Fat intake (12–60), mean (SD)^c^	27.9 (5.4)
None	33 (56.9)	Vegetable and fruit intake (5–21), mean (SD)^d^	13.5 (3.1)
1	8 (13.8)	Fibre intake (0–8), mean (SD)^d^	4.4 (1.5)
≥ 2	17 (29.3)	Physical activity	
Living situation		Total SQUASH score, mean (SD)	4745 (3923)
Alone	13 (22.4)	Steps/day, mean (SD)	7170 (3799)
With parent(s)	10 (17.2)	Total hours/week, mean (SD)	28.9 (20.8)
With roommates	2 (3.4)	Compliance with WHO guideline	41 (70.7)
With partner	11 (19.0)		
With children	2 (3.4)		
With partner and children	20 (34.5)		

Abbreviations: CNS, central nervous system; SD, standard deviation; SQUASH, Short Questionnaire to Assess Health‐Enhancing physical activity.

^a^ Germ cell tumour (*n* = 2) and rhabdomyosarcoma (*n* = 2).

^b^ Multiple categories possible.

^c^ A lower score indicates a healthier intake.

^d^ A higher score indicates a healthier intake.

### 3.2 Effect on Lifestyle Behaviours

#### 3.2.1 Primary Outcome

The majority of participants decided on more comprehensive lifestyle goals that focused on both healthier dietary intake (83%) and increasing physical activity (85%). In addition, 57% of personal goals were aimed at losing weight. An overview of the lifestyle goals chosen by participants is provided in Appendix S4.

**FIGURE 3:**
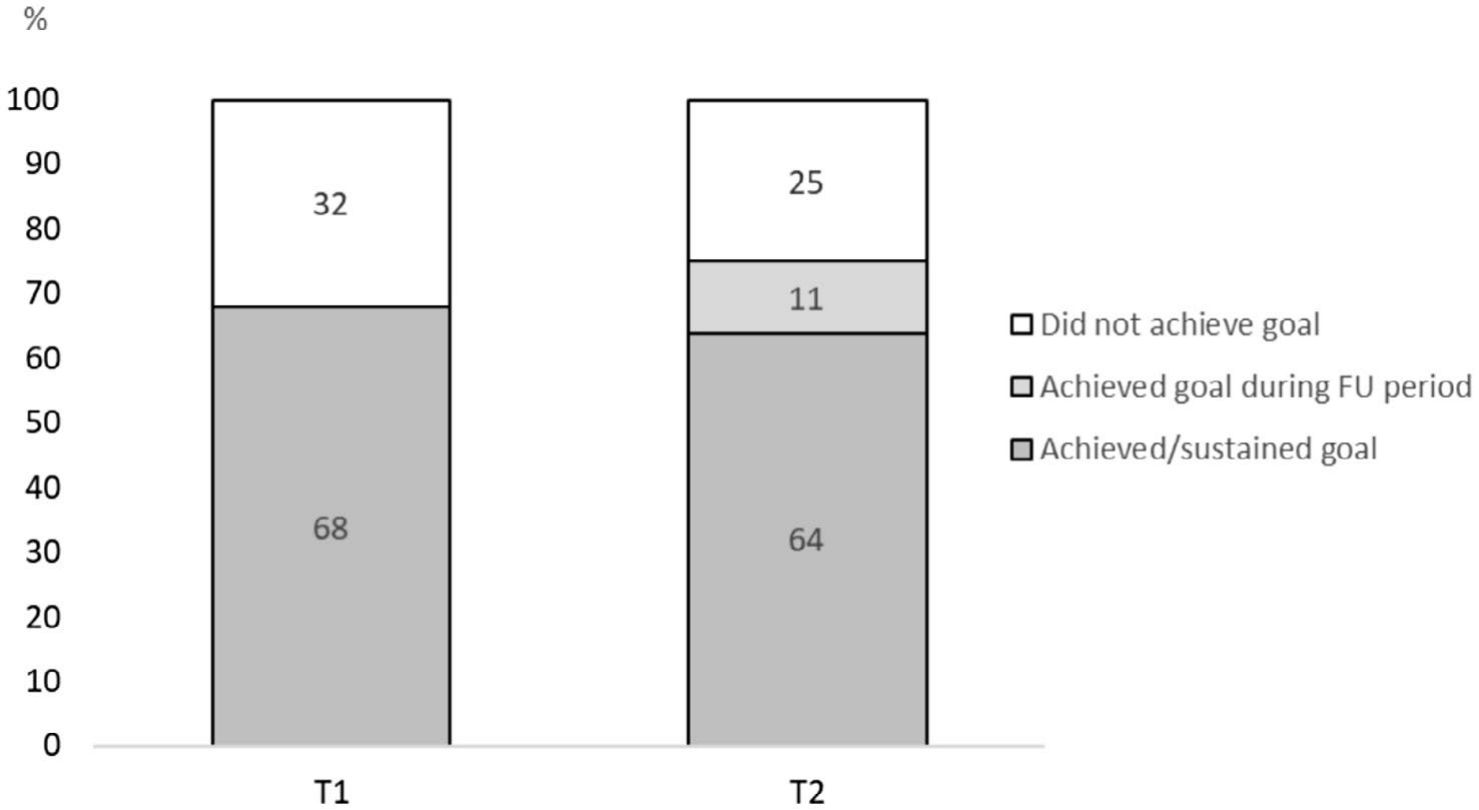
Personal lifestyle goals achieved and sustained. FU, follow‐up. T1: 68.4% of participants achieved their personal goal after 4 months of lifestyle coaching. T2: 64.2% of participants sustained their goal during another 4 months of follow‐up, 11.3% achieved their goal during the follow‐up period, and 24.5% never achieved their goal.

On average, participants completed 3.5 regular coaching sessions, with 8 participants completing 2, 17 participants completing 3, 25 participants completing 4 and 7 participants completing 5 regular coaching sessions. None of the participants opted for one or six regular sessions. By the end of the 4‐month coaching period (T1), 39/57 (68%) of participants achieved their predefined lifestyle goal (Figure 3). Of these 39 participants, 34 (92%) also sustained their goal during follow‐up (T2). Out of 18 participants who failed to attain their goal by T1, six reported having successfully reached their goal during the follow‐up period. Overall, 40/53 (76%) of participants achieved or sustained their goal at T2. Of those goals aimed at losing weight, healthier dietary intake and increased physical activity, 67%, 71% and 73% were achieved at T2, respectively.

#### 3.2.2 Secondary Outcomes

Between T0 and T2, significant improvements were observed in BMI (−0.5, SD = 1.4, *p* = 0.03) and dietary intake; the overall dietary intake score improved (−0.6, SD = 1.2, *p* < 0.01), fat intake decreased (−3.3, SD = 5.7, *p* = 0.001) and vegetable and fruit intake increased (1.1, SD = 2.6, *p* = 0.01). No significant difference was observed in fibre intake. In addition, the total SQUASH physical activity score increased by 1080 points (SD = 3248, *p* = 0.03) and the amount of time spent on physical activity increased by 7.7 h per week (SD = 20.9, *p* < 0.01) (Table 2). The mean number of steps per day, measured by the accelerometer, increased non‐significantly by 672 steps (SD = 3719). Results describing the change in self‐efficacy and self‐management skills are reported in Appendix S3.

**TABLE 2:** Changes in secondary outcome measures.

Outcome	T0 (*n* = 58)	T1 (*n* ~ 43)	T2 (*n* ~ 42)	Mean ΔT0–T2^a^	SD	Median (IQR)	*p* ^b^
	Mean (SD)/*n* (%)						
Body mass^c^							
BMI (kg/m^2^)	30.5 (4.9)	29.8 (4.8)	29.5 (4.7)	−0.5	1.4	0.0 (−0.3 to 1.3)	0.03
Weight (kg)	89.2 (17.7)	86.6 (16.3)	86.2 (16.7)	−1.3	3.9	0.0 (−3.3 to 1.0)	0.04
Dietary intake							
Overall diet score (3–9)^c^	6.5 (1.5)	5.9 (1.3)	5.6 (1.5)	−0.6	1.2	−1.0 (−1.0 to 0.0)	< 0.01^d^
Fat intake (12–60)^c^	27.9 (5.4)	24.0 (5.4)	24.0 (5.3)	−3.3	5.7	−3.0 (−7.25 to 3.0)	0.001^d^
Vegetable and fruit intake (5–21)^e^	13.5 (3.1)	14.3 (3.3)	15.2 (3.3)	1.1	2.6	1.0 (−1.0 to 3.0)	0.01^d^
Fibre intake (0–8)^e^	4.4 (1.5)	4.1 (1.6)	4.8 (1.5)	0.3	1.3	0.5 (−0.3 to 1.0)	0.09^f^
Physical activity							
SQUASH score, total	4745 (3923)	5931 (3934)	6044 (5115)	1081	3248	745 (−535 to 3520)	0.03^d^
Steps/day	7170 (3799)	7936 (3329)	7517 (3494)	672	3719	708 (−1423 to 2797)	0.27
Total hours/week	28.9 (20.8)	35.3 (22.2)	37.7 (28.7)	7.7	20.9	3.8 (−0.3 to 20.2)	< 0.01^d^
Compliance with WHO guideline, *n* (%)	41 (70.7)	37 (82.2)	32 (74.4)	7.5^f^	N/A	N/A	< 0.01

Abbreviations: SD, standard deviation; IQR, interquartile range; SQUASH, Short Questionnaire to Assess Health‐Enhancing physical activity.

^a^ Complete case analysis (*n* ~ 42).

^b^ *p*‐value from paired samples *t*‐test (normally distributed variables), Wilcoxon signed‐rank test (non‐normally distributed variables) or Fisher's exact test (compliance with WHO guideline [12]), T2 vs. T0.

^c^ A lower score indicates a healthier intake.

^d^ Data not normally distributed.

^e^ A higher score indicates a healthier intake.

^f^ Difference in percentage for complete cases (*N* = 40).

Finally, mixed modelling analysis showed improvements in BMI (estimate = −0.5, 95% CI = −0.8 to −0.1, *p* = 0.01), dietary intake (estimate = −0.5, 95% CI = −0.8 to −0.1, *p* < 0.01) and total SQUASH score (back‐transformed estimate = 1.3, 95% CI = 1.0–1.8, *p* = 0.02) at T2 compared to baseline (Table 3). The back‐transformed estimate for the total SQUASH score should be interpreted as a multiplicative factor (1.3 times higher SQUASH score). Findings describing predictors for greater improvements in BMI, physical activity and diet are reported in Appendix S3.

**TABLE 3:** Linear mixed modelling analysis: BMI, physical activity and dietary intake.

Determinants	BMI (kg/m^2^)		Physical activity (total SQUASH score^b^)		Dietary intake (overall diet score)^c^	
	Estimate (95% CI)	*p*	Estimate (95% CI)	*p*	Estimate (95% CI)	*p*
Intervention effect^a^						
T1	−0.7 (−1.1 to −0.3)	< 0.01	1.4 (1.1–1.9)	< 0.01	−0.5 (−0.9 to −0.2)	< 0.01
T2	−0.5 (−0.8 to −0.1)	0.01	1.3 (1.0–1.8)	0.02	−0.5 (−0.8 to −0.1)	0.01

*Note:* BMI: this model was adjusted for centre, age, age at diagnosis, sex, late effects burden, number of coaching sessions and having a personal goal focused at healthier dietary intake. Physical activity: this model was adjusted for centre, age, age at diagnosis, sex, late effects burden, employment status and timing of questionnaire completion with respect to COVID‐19 restrictions. Dietary intake: this model was adjusted for centre, age, age at diagnosis, sex, late effects burden and confidence in the ability to sustain a healthy lifestyle.

Abbreviations: BMI, body mass index, CI, confidence interval.

^a^ Versus baseline.

^b^ Back‐transformed estimate, to be interpreted as a multiplicative factor. Each model included a random intercept and an unstructured covariance matrix.

^c^ A lower score indicates a healthier intake.

### 3.3 Predictors for Goal Attainment and Sustainability

Multivariable logistic regression analysis revealed that participants with a lower BMI (OR 1.5; 95% CI, 1.1–2.1, *p* = 0.01) and fewer depressive feelings (OR 2.7; 95% CI, 1.2–6.1, *p* = 0.02) at baseline had a higher odds of achieving and sustaining their goals after 8 months. This model adjusted for centre, age, age at primary cancer diagnosis, sex, educational attainment and late effects burden. Due to the presence of few patients in some categories, a second model was estimated without adjustment for centre and educational level. Results were similar. Participants' age, age at diagnosis, sex and the number of attended coaching sessions were not associated with goal attainment and sustainability.

## 4 Discussion

Findings from this study suggest that the PCFU Lifestyle intervention successfully supports CAYA cancer survivors in making sustainable lifestyle changes. These results are in line with findings from two controlled trials conducted in diabetic patients, where one demonstrated significant reductions in BMI and haemoglobin A1c (HbA1c) after a 3‐month telemedicine‐based lifestyle coaching programme, and the other reported improved HbA1c and dietary intake after 6 months of telephone‐based lifestyle coaching [20, 21]. Furthermore, this study revealed that lifestyle coaching is likely to be more effective in CAYA cancer survivors who present with a lower BMI and fewer depressive feelings at baseline. These findings also align with existing evidence, which illustrates the compounding challenge of losing weight when having a higher BMI [22, 23]. In addition, depression has been previously identified as a predictor of poor dietary choices and physical inactivity in survivors of CAYA cancer [24, 25].

To the best of the authors' knowledge, this study is the first to assess one‐on‐one lifestyle coaching in the CAYA cancer survivor population. In addition, the concept of person‐centred care, on which the PCFU Lifestyle intervention was based, is what makes this intervention unique. By tailoring the intervention to accommodate individual needs and circumstances, personal barriers for sustainable health behaviour change can be more effectively addressed and facilitators can be exploited [26]. Moreover, by putting the primary focus on working towards a personalised lifestyle goal set through shared‐decision making, the diversity of participants' health priorities and capabilities is recognised. This person‐centred, tailored approach will likely increase the individual relevance of the intervention and could improve motivation, adherence and compliance [27].

In addition to the personalised approach, the PCFU Lifestyle intervention addresses the specific needs of survivors of CAYA cancers through educating lifestyle coaches in cancer survivorship and the specific barriers and facilitators that survivors experience in adopting healthier behaviours [28]. In addition, in case of complications, a direct line of communication exists between the lifestyle coach and the survivor's care provider or physiotherapist. That said, the intervention may also hold promise for other (cancer) patient populations. To tailor the intervention to these subgroup's needs and preferences, barriers and facilitators of adopting and sustaining healthy lifestyle behaviours in these populations should be investigated. Subsequently, lifestyle coaches should be educated on how these factors can be effectively used for supporting these populations.

This study has many strengths, such as the comprehensive assessment of comparability between questionnaire responders and non‐responders, which indicated a low risk of non‐response bias across all outcomes, except dietary intake. In addition, data were collected at three different time points, including 4 months of follow‐up time, providing insight into the efficacy of the PCFU Lifestyle intervention not only in the short term but also in the long term. Furthermore, due to the use of self‐reported outcomes, there was no need for participants to visit the outpatient clinic for data collection (e.g., to measure weight), which resulted in low attrition. Lastly, physical activity was measured using both a self‐reported questionnaire and an accelerometer to obtain a more comprehensive evaluation.

We should also acknowledge several limitations, which emphasise the need for further study in this area. First, this was a single‐arm study, which precluded a direct comparison with usual care. Moreover, we made use of self‐reported outcome measures, which may have introduced social desirability (e.g., feeling the obligation to report desirable outcomes) and recall biases. In particular for the small improvement found in BMI (−0.5 kg/m^2^), we should consider that these effects could have been caused by misreporting in self‐reported body weight, rather than the intervention effect. Furthermore, personal lifestyle goals were not always formulated in a manner that allowed for easy verification of their attainment, and although the Yamax Digiwalker was proven a reliable measuring instrument for scientific research [16], some participants in this study reported temporary interruptions in the smooth functioning of this device. For this reason, as well as the accelerometer's inability to capture certain physical activities such as water sports, rowing or fitness exercises, only self‐reported physical activity data were used for the multivariable analysis.

Future research should prioritise assessing the intervention's effectiveness using a randomised study design, comparing this intervention to usual care. Furthermore, participants should choose from a range of predefined lifestyle goals that are easily measurable and verifiable by an independent researcher, either during home visits or an additional visit to the outpatient clinic. A future study should also make use of more objectively assessed outcome measures, perhaps also including metabolic risk factors, physiological measures and cardiovascular outcomes, and should investigate whether the combination of online lifestyle coaching with other elements such as health education and peer support will improve results. In addition, as family history with a healthy lifestyle and their support may influence participation in physical activities and dietary intake, it is recommended for future studies to also collect and incorporate these data [29]. Lastly, although even small improvements in BMI can hold clinical significance, substantial improvements in health outcomes typically emerge from a 2%–5% reduction or more [30, 31]. In this study, a weight reduction of 1.5% was observed, and so it will be important to assess whether the PCFU eHealth lifestyle intervention will have a larger impact on BMI over a longer period of time. While it is hoped that participants will have gained the knowledge and tools necessary to further improve and maintain their lifestyle behaviours for years to come, they may also face the temptation to revert to old patterns.

Findings from this study are promising and support further study and implementation of online lifestyle coaching into clinical practice. Consequently, a replication manual including all necessary information and materials upon implementation of the PCFU Lifestyle intervention will be made freely available on the PanCare website (www.pancare.eu). As lifestyle coaching may be less effective in CAYA cancer survivors presenting with higher BMIs, referral for more extensive lifestyle support may be needed, such as a combined lifestyle intervention spanning approximately 1–2 years. In case of severe depressive symptoms, it will be important to prioritise mental health and emotional well‐being before embarking on an intensive lifestyle programme. However, if depressive symptoms are minimal, participation in the PCFU Lifestyle intervention could still be beneficial and may even help alleviate feelings of depression [32, 33]. Additional guidance on lifestyle support in CAYA cancer survivors will soon be provided by the International Late Effects of Childhood Cancer Guideline Harmonisation Group, who are currently developing a clinical practice guideline for health promotion (unpublished manuscript).

In conclusion, findings from this study suggest that the PCFU Lifestyle intervention successfully supports CAYA cancer survivors in making lifestyle changes that are sustainable for at least 4 months. Although future research will be needed to more accurately determine effect sizes, positive results from this study warrant the integration of online lifestyle support into care, which could greatly improve survivors' health outcomes and quality of life.

## Author Contributions

**Selina R. van den Oever:** conceptualization (equal), data curation (equal), formal analysis (equal), investigation (equal), methodology (equal), project administration (equal), visualization (equal), writing – original draft (equal). **Eline Bouwman:** conceptualization (equal), investigation (equal), methodology (equal), visualization (equal), writing – original draft (equal). **Helena J. H. van der Pal:** conceptualization (equal), formal analysis (equal), investigation (equal), methodology (equal), supervision (equal), writing – original draft (equal). **Philippa C. Steensma:** data curation (equal), formal analysis (equal), investigation (equal), methodology (equal), writing – original draft (equal), writing – review and editing (equal). **Vera Araujo‐Soares:** conceptualization (equal), methodology (equal), writing – review and editing (equal). **Morven Brown:** conceptualization (equal), methodology (equal), writing – review and editing (equal). **Tomas Kepak:** conceptualization (equal), methodology (equal), writing – review and editing (equal). **Katerina Kepakova:** conceptualization (equal), methodology (equal), writing – review and editing (equal). **Marta Fiocco:** formal analysis (equal), methodology (equal), writing – review and editing (equal). **Lucy M. M. Fremouw:** investigation (equal), methodology (equal), writing – review and editing (equal). **Maria M. W. Koopman:** investigation (equal), writing – review and editing (equal). **Raphaële R. L. van Litsenburg:** methodology (equal), writing – review and editing (equal). **Patrick van der Torre:** investigation (equal), methodology (equal), writing – review and editing (supporting). **Joyce Wilbers:** investigation (equal), writing – review and editing (equal). **Roderick Skinner:** conceptualization (equal), methodology (equal), writing – review and editing (equal). **Leontien C. M. Kremer:** conceptualization (equal), formal analysis (equal), funding acquisition (equal), methodology (equal), supervision (equal), visualization (equal), writing – original draft (equal). **Jacqueline Loonen:** conceptualization (equal), formal analysis (equal), funding acquisition (equal), investigation (equal), methodology (equal), visualization (equal), writing – original draft (equal). **Saskia M. F. Pluijm:** conceptualization (equal), data curation (equal), formal analysis (equal), funding acquisition (equal), investigation (equal), methodology (equal), project administration (equal), supervision (equal), visualization (equal), writing – original draft (equal).

## Ethics Statement

This study was declared not to be subject to the Medical Research Involving Human Subjects Act (WMO) by the Research Ethics Committee CMO Arnhem‐Nijmegen in September 2020 (case number 2020‐6960). Therefore, no positive judgement was required from the CMO region Arnhem‐Nijmegen or another recognised medical ethical review committee for its implementation. Local research and ethics committees at the participating centres have granted their approvals for the conduction of this study (Research Ethics Committee Radboudumc and Clinical Research Centre Princess Máxima Centre).

## Consent

Informed consent procedures are described by Bouwman et al. [13].

## Conflicts of Interest

The authors declare no conflicts of interest.

## Supporting information


Appendix S1.–S4.

## Data Availability

Study participants did not consent to data sharing outside the PCFU consortium. Therefore, access to participant data is limited to national and international supervisory authorities. The study protocol can be made available to researchers upon request (please send an email to s.r.vandenoever-2@prinsesmaximacentrum.nl).
