# Supplementary material for: Efficacy of the PanCareFollowUp eHealth Lifestyle Intervention for Survivors of Childhood, Adolescent and Young Adult Cancer
Source: Cancer Med. 2025 Feb 28;14(5):e70694. doi: 10.1002/cam4.70694 (PMC11868983; doi:10.1002/cam4.70694)
Supplement: Supplementary file 1 — Appendix S1.–S4. [file CAM4-14-e70694-s001.docx]

**Appendix S1.** Members of the PanCareFollowUp Consortium

Leontien C.M. Kremer, Princess Máxima Centre for Paediatric Oncology, Heidelberglaan 25, 3584 CS, Utrecht, the Netherlands; Faculty of Medicine, Utrecht University and Utrecht Medical Centre, Universiteitsweg 98, 3584 CG Utrecht, the Netherlands

Helena J.H. van der Pal, Princess Máxima Centre for Paediatric Oncology, Heidelberglaan 25, 3584 CS, Utrecht, the Netherlands; PanCare, Jacobus Bellamylaan 16, 1401 AZ Bussum, the Netherlands

Renée L. Mulder, Princess Máxima Centre for Paediatric Oncology, Heidelberglaan 25, 3584 CS, Utrecht, the Netherlands

Saskia M.F. Pluijm, Princess Máxima Centre for Paediatric Oncology, Heidelberglaan 25, 3584 CS, Utrecht, the Netherlands

Rebecca J. van Kalsbeek, Princess Máxima Centre for Paediatric Oncology, Heidelberglaan 25, 3584 CS, Utrecht, the Netherlands

Selina R. van den Oever, Princess Máxima Centre for Paediatric Oncology, Heidelberglaan 25, 3584 CS, Utrecht, the Netherlands

E. A. M. (Lieke) Feijen, Princess Máxima Centre for Paediatric Oncology, Heidelberglaan 25, 3584 CS, Utrecht, the Netherlands

Lars Hjorth, Lund University, Skane University Hospital, Department of Clinical Sciences Lund, Paediatrics, Lasarettsgatan 40, 221 85 Lund, Sweden

Cecilia Follin, Lund University, Skane University Hospital, Department of Clinical Sciences Lund, Oncology, Lasarettsgatan 40, 221 85 Lund, Sweden

Lill Eriksson, Lund University, Skane University Hospital, Department of Clinical Sciences Lund, Oncology, Lasarettsgatan 40, 221 85 Lund, Sweden

Thomas Relander, Lund University, Skane University Hospital, Department of Clinical Sciences Lund, Oncology, Lasarettsgatan 40, 221 85 Lund, Sweden

Jacob Engellau, Lund University, Skane University Hospital, Department of Clinical Sciences Lund, Oncology, Lasarettsgatan 40, 221 85 Lund, Sweden

Karolina Bogefors, Lund University, Skane University Hospital, Department of Clinical Sciences Lund, Oncology, Lasarettsgatan 40, 221 85 Lund, Sweden

Anna Sällfors Holmqvist, Lund University, Skane University Hospital, Department of Clinical Sciences Lund, Paediatrics, Lasarettsgatan 40, 221 85 Lund, Sweden

Riccardo Haupt, Epidemiology and Biostatistics Unit and DOPO clinic, IRCCS Istituto Giannina Gaslini, Via G. Gaslini, 5, 16147 Genoa, Italy

Monica Muraca, Epidemiology and Biostatistics Unit and DOPO clinic, IRCCS Istituto Giannina Gaslini, Via G. Gaslini, 5, 16147 Genoa, Italy

Brigitte Nicolas, Epidemiology and Biostatistics Unit and DOPO clinic, IRCCS Istituto Giannina Gaslini, Via G. Gaslini, 5, 16147 Genoa, Italy

Francesca Bagnasco, Epidemiology and Biostatistics Unit and DOPO clinic, IRCCS Istituto Giannina Gaslini, Via G. Gaslini, 5, 16147 Genoa, Italy

Marina Benvenuto, Epidemiology and Biostatistics Unit and DOPO clinic, IRCCS Istituto Giannina Gaslini, Via G. Gaslini, 5, 16147 Genoa, Italy

Anna Aulicino, Epidemiology and Biostatistics Unit and DOPO clinic, IRCCS Istituto Giannina Gaslini, Via G. Gaslini, 5, 16147 Genoa, Italy

Luca Laudisi, Epidemiology and Biostatistics Unit and DOPO clinic, IRCCS Istituto Giannina Gaslini, Via G. Gaslini, 5 – 16147 Genoa, Italy

Vera Araujo-Soares, Center for Preventive Medicine and Digital Health, Theodor-Kutzer-Ufer 1-3
68167 Mannheim, Germany

Tomas Kepak, International Clinical Research Center, St. Anne's University Hospital Brno, , Pekařská 53, Brno 656 91, Czech Republic

Katerina Kepakova, International Clinical Research Center, St. Anne's University Hospital Brno, Pekařská 53, Brno 656 91, Czech Republic

Hana Hrstkova, International Clinical Research Center, St. Anne's University Hospital Brno, Pekařská 53, Brno 656 91, Czech Republic

Viera Bajciova, International Clinical Research Center, St. Anne's University Hospital Brno, Pekařská 53, Brno 656 91, Czech Republic

Marta Holikova, International Clinical Research Center, St. Anne's University Hospital Brno, Pekařská 53, Brno 656 91, Czech Republic

Lucie Strublova, International Clinical Research Center, St. Anne's University Hospital Brno, Pekařská 53, Brno 656 91, Czech Republic

Anne Uyttebroeck, Department of Oncology, Paediatric Oncology, KU Leuven, Department of Paediatric Haematology and Oncology, University Hospitals Leuven, Herestraat 49, 3000 Leuven, Belgium

Marleen Renard, Department of Paediatric Haematology and Oncology, University Hospitals Leuven, Herestraat 49, 3000 Leuven, Belgium

Sandra Jacobs, Department of Oncology, Paediatric Oncology, KU Leuven, Department of Paediatric Haematology and Oncology, University Hospitals Leuven, Herestraat 49, 3000 Leuven, Belgium

Heidi Segers, Department of Oncology, Paediatric Oncology, KU Leuven, Department of Paediatric Haematology and Oncology, University Hospitals Leuven, Herestraat 49, 3000 Leuven, Belgium

Maria van Helvoirt, Department of Paediatric Haematology and Oncology, University Hospitals Leuven, Herestraat 49, 3000 Leuven, Belgium

Charlotte Sleurs, Department of Paediatric Haematology and Oncology, University Hospitals Leuven, Herestraat 49, 3000 Leuven, Belgium

Jeanette Falck Winther, Childhood Cancer Research Group, Danish Cancer Society Research Centre, Strandboulevarden 49, 2100 Copenhagen, Denmark; Department of Clinical Medicine, Faculty of Health, Aarhus University and Aarhus University Hospital, Palle Juul-Jensens Boulevard 82, 8200 Aarhus, Denmark

Luzius Mader, Childhood Cancer Research Group, Danish Cancer Society Research Centre, Strandboulevarden 49, 2100 Copenhagen, Denmark; Institute of Social and Preventive Medicine, University of Bern, Mittelstrasse 43, 3012 Bern, Switzerland

Line Elmerdahl Frederiksen, Childhood Cancer Research Group, Danish Cancer Society Research Centre, Strandboulevarden 49, 2100 Copenhagen, Denmark

Elisabeth Anne Wreford Andersen, Statistics and Data Analysis, Danish Cancer Society Research Centre, Strandboulevarden 49, 2100 Copenhagen, Denmark

Marrieta Kokla, Childhood Cancer Research Group, Danish Cancer Society Research Centre, Strandboulevarden 49, 2100 Copenhagen, Denmark

Anja Krøyer, Childhood Cancer Research Group, Danish Cancer Society Research Centre, Strandboulevarden 49, 2100 Copenhagen, Denmark

Thomas Tjørnelund Nielsen, Childhood Cancer Research Group, Danish Cancer Society Research Centre, Strandboulevarden 49, 2100 Copenhagen, Denmark

Gisela Michel, University of Lucerne, Faculty of Health Sciences and Medicine, Alpenquai 4, 6005 Lucerne, Switzerland

Stefan Boes, University of Lucerne, Faculty of Health Sciences and Medicine, Alpenquai 4, 6005 Lucerne, Switzerland

Katharina Roser, University of Lucerne, Faculty of Health Sciences and MedicineAlpenquai 4, 6005 Lucerne, Switzerland

Jacqueline Loonen, Radboud University Medical Centre, Radboud Institute for Health Sciences, Department of Hematology, Geert Grooteplein Zuid 10, 6525 GA, Nijmegen, the Netherlands

Rosella Hermens, Radboud University Medical Centre, Radboud Institute for Health Sciences, Scientific Institute for Quality of Healthcare (IQ Healthcare), Geert Grooteplein 21, 6525 EZ, Nijmegen, the Netherlands

Irene Göttgens, Radboud University Medical Centre, Radboud Institute for Health Sciences, Department of Primary and Community Care, Geert Grooteplein 21, 6525 EZ, Nijmegen, the Netherlands

Eline Bouwman, Radboud University Medical Centre, Radboud Institute for Health Sciences, Department of Hematology, Geert Grooteplein Zuid 10, 6525 GA, Nijmegen, the Netherlands

Iridi Stollman, Radboud University Medical Centre, Radboud Institute for Health Sciences, Department of Hematology, Geert Grooteplein Zuid 10, 6525 GA, Nijmegen, the Netherlands

Adriaan Penson, Radboud University Medical Centre, Radboud Institute for Health Sciences, Department of Hematology, Geert Grooteplein Zuid 10, 6525 GA, Nijmegen, the Netherlands

Dionne Breij, Radboud University Medical Centre, Radboud Institute for Health Sciences, Department of Hematology, Geert Grooteplein Zuid 10, 6525 GA, Nijmegen, the Netherlands

Roderick Skinner, Newcastle University Centre for Cancer, Wolfson Childhood Cancer Research Centre, Herschel Building, Brewery Lane, Newcastle upon Tyne, NE1 7RU, United Kingdom; Great North Children’s Hospital, Royal Victoria Infirmary, Queen Victoria Road, Newcastle upon Tyne, NE1 4 LP, United Kingdom; Translational and Clinical Research Institute, Wolfson Childhood Cancer Research Centre, Herschel Building, Brewery Lane, Newcastle upon Tyne, NE1 7RU, United Kingdom

Morven C. Brown, Population Health Sciences Institute, Newcastle University, Sir James Spence Institute, Royal Victoria Infirmary, Queen Victoria Road, Newcastle upon Tyne, NE1 4LP, United Kingdom; Newcastle University Centre for Cancer, Wolfson Childhood Cancer Research Centre, Herschel Building, Brewery Lane, Newcastle upon Tyne, NE1 7RU, United Kingdom

Samira Essiaf, European Society for Paediatric Oncology, c/o BLSI, Clos Chapelle-aux-Champs 30, Bte 1.30.30, BE-1200 Brussels, Belgium

Anne Blondeel, European Society for Paediatric Oncology, c/o BLSI, Clos Chapelle-aux-Champs 30, Bte 1.30.30, BE-1200 Brussels, Belgium

William Sciberras, European Society for Paediatric Oncology, c/o BLSI, Clos Chapelle-aux-Champs 30, Bte 1.30.30, BE-1200 Brussels, Belgium

Giorgia Manuzi, European Society for Paediatric Oncology, c/o BLSI, Clos Chapelle-aux-Champs 30, Bte 1.30.30, BE-1200 Brussels, Belgium

Joke Korevaar, Netherlands Institute for Health Services Research (Nivel), P.O. Box 1568, 3500 BN Utrecht, the Netherlands

Mieke Rijken, Netherlands Institute for Health Services Research (Nivel), P.O. Box 1568, 3500 BN Utrecht, the Netherlands; University of Eastern Finland, Department of Health and Social Management, P.O. Box 1627, FI-70211 Kuopio, Finland

Anita Kienesberger, Childhood Cancer International – Europe, Lerchenfelderstraße 74, Stiege 3/Top 2, 1080 Vienna, Austria

Jaap den Hartogh, Princess Máxima Centre for Paediatric Oncology, Heidelberglaan 25, 3584 CS, Utrecht, the Netherlands; Dutch Childhood Cancer Organization (Vereniging Kinderkanker Nederland), De Bilt, The Netherlands

Hannah Gsell, Childhood Cancer International – Europe, Lerchenfelderstraße 74, Stiege 3/Top 2, 1080 Vienna, Austria

Carina Schneider, Childhood Cancer International – Europe, Lerchenfelderstraße 74, Stiege 3/Top 2, 1080 Vienna, Austria

Edit Bardi, St. Anna Children’s Hospital, Kinderspitalgasse 6, Vienna, 1090, Austria, Kepler University Clinic, Department of Pediatric and Adolescent Medicine, Krankenhausstraße 26-30, Linz, 4020, Austria

Jeroen te Dorsthorst, PanCare, Jacobus Bellamylaan 16, 1401 AZ Bussum, the Netherlands

**Appendix S2.** Power calculations.

Power calculations were performed using G*Power version 3.1.9.7. For the primary outcome, it was hypothesised that 60% of participants would achieve their goal at T1, while 30% was considered acceptable. This corresponded to an effect size (Cohen's h) of 0.6. Using a binomial test with an alpha level of 0.05, a sample size of 20 participants was determined to provide 80% power.

For the secondary outcomes of BMI, dietary intake, and physical activity, a two-tailed, matched pairs t-test was used to determine the required sample size to detect medium effects. Based on existing scientific evidence, the effect size (Cohen's dz) was set at 0.4 for all three outcomes. The alpha level was set at 0.05 and the desired power at 80%, resulting in a minimum sample size of 52 participants.

**Appendix S3.** Extended analysis.

In this study, we aimed to assess the PCFU Lifestyle intervention’s impact on self-efficacy and self-management skills. These are two outcomes that the concept of person-centred care, on which the intervention is based, is expected to improve. In addition, we aimed to identify predictors for greater improvements in secondary outcomes (BMI, physical activity and dietary intake) over time. To improve readability and understanding of the main research paper, we present the results of these analyses in this appendix.

**Self-efficacy**

Self-efficacy was assessed using the General Self-Efficacy (GSE) Scale^15^. Scores at baseline did not differ between questionnaire completers and non-responders. The mean differences between T2 and T0 (∆T2-T0) were examined for complete cases using Wilcoxon signed-rank test. Compared to baseline, participants reported higher self-efficacy scores at T2 (1.1, SD=5.9, p=0.03) (Table S1).

**Self-management**

We made use of the Self-Management Screening (SeMaS) tool^16^ to assess self-management skills in different domains. This questionnaire was developed for clinical care and has not been validated for scientific research. Upon analysis of the results, we encountered issues in calculating meaningful sum scores. Consequently, we decided to only include the following domains in our analysis: 1) the burden of late effects on participants’ daily life (score 0 to 10, no burden to unbearable burden), 2) willingness to self-manage (score 0 to 3, not willing to willing), 3) perceived control over health (score 0 to 6, no control to a lot of control), 4) confidence in the ability to sustain a healthy lifestyle (score 0 to 6, low confidence to high confidence), 5) feelings of anxiety (score 0 to 8, never to often), 6) and feelings of depression (score 0 to 6, never to often). Although participants with severe depressive symptoms were excluded from this study, we decided to include this last domain as mild feelings of depression may still impact CAYA cancer survivors’ ability to self-manage. While domains 2, 3 and 4 were included in this extended analysis as outcome variables, we did not expect an effect of the PCFU Lifestyle intervention on domains 1, 5 and 6. Hence, these domains were only considered as covariates in the main analyses.

| **Table S1.** Changes in self-efficacy and self-management skills. | | | | | | | | | | | |
| --- | --- | --- | --- | --- | --- | --- | --- | --- | --- | --- | --- |
| Outcome | T0 (n=58) | T1 (n~43) | | T2 (n~42) | | Mean ΔT0-T2^a^ | SD | | Median (IQR) | | p^b^ |
|  | Mean (SD) / n (%) | | | | |  | |  | |  |  |
| Self-efficacy |  | |  | |  |  |  | |  | |  |
| GSE score (10-40) | 30.1 (4.5) | 30.8 (5.1) | | 31.1 (4.8) | | 1.1 | 5.9 | | 1.0 (-1.0 – 3.3) | | 0.03 |
| Self-management |  |  | |  | |  |  | |  | |  |
| Willingness to self-manage (0-3) | 2.7 (0.5) | 2.5 (0.6) | | 2.5 (0.6) | | -0.2 | 0.5 | | 0.0 (0.0 – 0.0) | | 0.04 |
| Perceived control over health (0-6) | 3.7 (0.9) | 3.7 (0.9) | | 3.7 (0.9) | | 0.0 | 1.1 | | 0.0 (-1.0 – 1.0) | | 0.92 |
| Confidence in ability to sustain a healthy lifestyle (0-6) | 4.4 (0.9) | 4.6 (1.1) | | 5.0 (1.0) | | 0.5 | 0.9 | | 0.0 (0.0 – 1.0) | | <0.01 |

Regarding the forementioned six self-management domains, no differences in baseline scores were revealed between questionnaire completers and non-responders. The mean differences between T2 and T0 (∆T2-T0) for domains 2, 3 and 4 were examined for complete cases using a Wilcoxon signed-rank test. Willingness to self-manage decreased (-0.2, SD=0.5, p=0.04) while participants’ confidence in the ability to sustain a healthy lifestyle increased (0.5, SD=0.9, p<0.01). No significant differences were observed in participants’ perceived control over their health.

Although the coaching sessions aimed to enhance self-management skills, the slight yet significant decrease in survivors’ willingness to self-manage may suggest that receiving lifestyle support can reduce their perceived need - and consequently their motivation - to manage their own health independently. Potentially, this could pose a risk to the sustainability of the intervention’s effects long term, which should be investigated in future studies with longer follow-up. That said, the mean score for this variable still equalled 2.5 out of 3, suggesting high willingness to self-manage overall.

Abbreviations: SD, standard deviation, IQR, interquartile range, GSE, general self-efficacy. ^a^Complete case analysis (n~42). ^b^P-value from Wilcoxon signed-rank test (non-normally distributed variables), T2 vs T0.

**Predictors for greater improvements in BMI, physical activity and dietary intake**

To identify predictors for greater improvements in BMI, physical activity, and dietary intake over time, potential interactions between the intervention effect and age, sex, educational attainment, and late effects burden were assessed. In addition, we investigated whether having a predefined lifestyle goal focused on either weight loss, healthier diet, or physical activity also resulted in greater improvements in their corresponding outcome, and whether a greater number of coaching sessions was also associated with greater improvements. In this extended analysis, significant interaction effects (time*variable) were added to the multiple linear mixed models used for the main analysis. Significant interactions indicated a different intervention effect in one subgroup versus another.

The extended mixed modelling analysis involving interaction terms revealed an association between higher educational attainment and a greater reduction in BMI after four months of follow-up (estimate=-0.7, 95% CI =-1.2–-0.2, p<0.01). Age, sex, late effects burden, and having a personal goal aimed at weight loss were not associated with a greater reduction in BMI over time. In addition, more coaching sessions did not result in greater improvements in BMI.

Compared to participants with a differently focused goal, participants whose personal goal was aimed at healthier eating habits reported greater improvements in dietary intake at T1 (estimate=-1.1, 95% CI = -1.9–-0.3, p=0.01) but not T2. Moreover, after follow-up, greater improvements in diet were observed in participants of older age (estimate=-0.03, 95% CI = 0.06–0.00), p=0.03). Age, sex, educational attainment, late effects burden, and the number of coaching sessions were not associated with greater dietary improvements. No predictors could be identified for greater improvements in physical activity over time.

**Appendix S4.** Personal lifestyle goals, defined during the intake session.

| **Personal lifestyle goals** | | ***n*** |
| --- | --- | --- |
| 1 | Lose 5 kg. | 14 |
| 2 | Lose 2 kg. | 4 |
| 3 | Lose 3 kg. | 4 |
| 4 | Eat 3 healthy meals a day. | 3 |
| 5 | Lose 4 kg. | 2 |
| 6 | Lose 2,5 kg. | 1 |
| 7 | Lose 2 kg and climb the stairs at work without getting out of breath. | 1 |
| 8 | 3 healthy meals a day (and would like to lose 4 kg). | 1 |
| 9 | Eat breakfast every day. | 1 |
| 10 | Run 5 km. | 1 |
| 11 | Take 42.000 steps/week. | 1 |
| 12 | Run 5 km twice/week and lose 5 kg. | 1 |
| 13 | Climb the 3 stairs at work without getting out of breath. | 1 |
| 14 | Structure the day, go to bed at 21.30 hr and sleep at 23.00 hr. Breakfast at  9.00 hr, lunch at 12.30 hr and dinner at 18.00 hr. | 1 |
| 15 | Feeling fitter and more energetic. | 1 |
| 16 | Become more aware of importance of healthy diet, change behaviours for sustainable results. | 1 |
| 17 | Lose weight. | 1 |
| 18 | Healthier dietary intake en more physical activity. Lower cholesterol levels. | 1 |
| 19 | 8000 steps per day (Mondays – Fridays), sufficient intake of protein and carbohydrates. | 1 |
| 20 | Limit intake of glasses of coke, more exercise. | 1 |
| 21 | Improve lifestyle behaviours, including dietary intake and physical activity. | 1 |
| 22 | Make conscious choices in diet, more physical activity, walk 1 hr every day and lose 5 kg. | 1 |
| 23 | 30 minutes of moderate intensity physical activity for 5 days/week. | 1 |
| 24 | (Moderate intensity) physical activity 3 times/ week. | 1 |
| 25 | Do sports 3 times/week. | 1 |
| 26 | Take 25.000 steps/week. | 1 |
| 27 | Lose 5 kg, increase muscle mass and reduce fat mass. | 1 |
| 28 | Take 10.000 steps/day, for 4 days/week. Waterpolo practice 2 times/week. | 1 |
| 29 | Take 6.000-8.000 steps/day and improve dietary intake. | 1 |
| 30 | Take 5.000 steps/day and run for ≥30 minutes twice/week. |  |
| 31 | Take 10.000 steps/day, and structurally integrate race cycling and swimming sessions. Lose  20 kg. | 1 |
| 32 | Take 8.000 steps/day. | 1 |
| 33 | Take 10.000 steps/day, at least 1 hr of physical activity/day. | 1 |
| 34 | Take 10.000 steps/day. | 1 |
| 35 | Healthier dietary intake and feeling fitter, hopes to lose weight. | 1 |
| 36 | Personal lifestyle goal left undecided (dropout after intake session). | 1 |
